# Paediatric Subanalysis of TSUBASA, Assessing Physical Activity, Bleeding, Quality of Life and Safety in People with Haemophilia A Receiving Emicizumab

**DOI:** 10.1055/a-2781-8278

**Published:** 2026-01-21

**Authors:** Keiji Nogami, Kagehiro Amano, Akihiro Sawada, Azusa Nagao, Chiai Nagae, Masanori Nojima, Nobuaki Suzuki, Mika Kawano, Tomomi Shimura, Yoshimasa Sugao, Teruhisa Fujii

**Affiliations:** 1 Department of Pediatrics, Nara Medical University, Nara, Japan; 2 Department of Laboratory Medicine, Tokyo Medical University, Tokyo, Japan; 3 Department of Respiratory Medicine and Hematology, Hyogo Medical University, Hyogo, Japan; 4 Department of Hematology and Oncology, Kansai Medical University Hospital, Osaka, Japan; 5 Department of Blood Coagulation, Ogikubo Hospital, Tokyo, Japan; 6 Department of Pediatrics, St. Marianna University School of Medicine, Kanagawa, Japan; 7 Center for Translational Research/Division of Advanced Medicine Promotion, Institute of Medical Science, University of Tokyo, Tokyo, Japan; 8 Department of Transfusion Medicine, Nagoya University Hospital, Aichi, Japan; 9 Medical Science, Chugai Pharmaceutical Co., Ltd., Tokyo, Japan; 10 Division of Transfusion Medicine/Hemophilia Treatment Center, Hiroshima University Hospital, Hiroshima, Japan

**Keywords:** exercise, Japan, children, adolescents, prophylaxis

## Abstract

**Introduction:**

Limited data are available on the relationship between bleeding outcomes and physical activity, and the quality of daily life (QoL), in children with haemophilia A (HA) receiving emicizumab prophylaxis.

**Aim:**

TSUBASA evaluated physical activity, bleeding events, safety, and QoL in Japanese people with HA initiating emicizumab prophylaxis. This paper reports the results from the final analysis, focusing on children and adolescents with HA without factor VIII inhibitors, and their caregivers.

**Methods:**

TSUBASA was a prospective, multicentre, observational study conducted across 50 medical institutions in Japan. Participants received emicizumab for 97 weeks. Bleeding events and physical activity data were obtained using an electronic patient-reported outcomes application; activity intensity was collected by wearable activity trackers worn over five 8-day monitoring periods. Adverse events (AEs) were documented on the electronic case report form and QoL was assessed using questionnaires.

**Results:**

A total of 46 participants aged <18 years were enrolled; most (84.8%) had severe HA. Over a median observation period of 674 days (quartile 1–quartile 3: 665–690), the mean annualized bleed rate was 0.86 (standard deviation: 1.26). In all Twenty-six participants experienced 66 AEs, of which 2 were injection-site reactions deemed related to emicizumab. J-KIDSCREEN-52 questionnaire scores were maintained from baseline onwards. Of the completed caregiver questionnaires ( *n* = 32), 43.8% reported increased activity and 56.3% reported unchanged activity. Additionally, 56.3% reported decreased anxiety about bleeding and 37.5% reported unchanged anxiety about bleeding. A total of 172 events of physical activity were recorded by 19 participants; 44 were high risk, 70 were moderate risk, and 42 were low risk. One activity-related traumatic bleed resulting from the impact of a basketball occurred, and more than 25 different types of physical activity were performed without bleeding.

**Conclusion:**

Children and adolescents with HA who receive prophylaxis with emicizumab may be able to engage in consistent physical activity, with a low risk of experiencing bleeds. Additionally, the questionnaire responses from caregivers on physical activity and caregiver experience provide rare insights into the real-world impact of emicizumab prophylaxis. These findings present a more comprehensive view of the benefits of emicizumab. No new safety signals were observed and QoL was maintained for 2 years after emicizumab initiation.

## Introduction

People with haemophilia A (PwHA) were historically discouraged from participating in physical activity due to associated risk of increased bleeding, which could lead to haemophilic arthropathy and joint damage. 1 However, with early prophylaxis now endorsed by the World Federation of Hemophilia as the standard of care for people with moderate or severe haemophilia, physical activity is recommended due to its recognized health benefits, including improvements in joint, bone, and muscle health. 2 3 4 These improvements are associated with reduced pain and disability, which may lead to greater involvement in recreational activities, school, and work. 3 Emicizumab is a recombinant, bispecific monoclonal antibody approved for use in PwHA with or without factor (F) VIII inhibitors. 5 6 7 The half-life of emicizumab is approximately 30 days, allowing for adaptable subcutaneous maintenance dosing. 7 8 9 10 11 Emicizumab has revolutionized prophylaxis in children with haemophilia A (HA), in whom FVIII replacement therapy can be challenging due to the frequent intravenous infusions required and issues with intravenous access. 3 Efficacy and safety of emicizumab in children and infants have been illustrated in clinical trials. 9 12 13 14 Emicizumab has also been associated with clinically meaningful improvements in health-related quality of life (QoL) outcomes in both children and adults. 7 14 15 16 17 It is expected that the low bleed rates reported with emicizumab will translate to increased engagement in physical activity, but data on physical activity in children with HA receiving emicizumab are limited.

TSUBASA (UMIN-CTR ID: UMIN000037448) was designed to evaluate physical activity, bleeding events, safety, and QoL, in Japanese PwHA initiating emicizumab prophylaxis. Interim analyses found that participants experienced minimal bleeding associated with physical activity, and provided further evidence of the effectiveness and favourable safety profile of emicizumab. 18 19 Here, we report results from the final analysis of the aforementioned TSUBASA study, focusing on outcomes in children and adolescent populations with HA without FVIII inhibitors.

## Methods

### Study Design and Participants

TSUBASA was a prospective, multicentre, observational study conducted across 50 participating institutions in Japan. Participants with congenital HA without FVIII inhibitors who had emicizumab selected as the most appropriate treatment were enrolled. Full inclusion and exclusion criteria have been previously published. 20 This analysis focused on participants <18 years old enrolled in the study, examining outcomes in this important age group. Emicizumab was administered according to approved dosing regimens: a loading dose of 3 mg/kg QW for 4 weeks, followed by maintenance doses of 1.5 mg/kg QW, 3 mg/kg Q2W, or 6 mg/kg Q4W. Treatment continued for 97 weeks after the first dose, or until it was considered clinically inappropriate.

### Objectives

TSUBASA was designed to evaluate physical activity, bleeding events, safety, and QoL in Japanese PwHA initiating emicizumab prophylaxis. The main objective of this analysis is to report these outcomes in children and adolescents (participants aged <18 years) enrolled in the TSUBASA study, particularly annualized bleed rates (ABRs) and proportions of participants with zero bleeding events. This analysis had a particular focus on children aged <2 years old.

Efficacy endpoints related to bleeding included ABRs for treated bleeds and proportions of paediatric participants with zero treated bleeds. Efficacy endpoints documenting the relationship between physical activity status and bleeds included patient-reported physical activity status, patient-reported status of bleeds attributable to physical activity, and data on physical activity (participants ≥6 years old). The International Physical Activity Questionnaire 21 (IPAQ, participants aged ≥6 years old) assessed the intensity and amount of physical activity performed during the study. Safety endpoints included evaluation of adverse events (AEs) and serious adverse events (SAEs), and their relationship with emicizumab, and development of FVIII inhibitors. QoL was assessed with a number of tools. The Japanese version of the KIDSCREEN-52 questionnaire (J-KIDSCREEN-52, 22 participants aged 6–15 years old) assessed physical well-being, psychological well-being, mood and emotions, self-perception, autonomy, parent relationship and home life, social support and peers, school environment, social acceptance (bullying), and financial resources. A questionnaire-based survey on daily life completed by participants aged ≥6 years old and caregivers collected information regarding activity, frequency of activity, motivation for work/school, and anxiety about bleeding.

### Data Collection

Treated bleeds and treatments were recorded using the electronic patient-reported outcomes (ePRO) mobile application by participants or their caregivers throughout the study. Participants or their caregivers also used the ePRO to record whether they believed bleeds were attributable to physical activity during the five physical activity monitoring periods (see below).

Quantitative data on physical activity were collected using CentrePoint Insight Watches; these medical-grade wearable activity trackers are optimized to capture total movement, moderate-to-vigorous physical activity, non-sedentary time step count, and energy expenditure in clinical research. 23 To aid compliance, as wearable trackers were not common in Japan when the study commenced, activity trackers were only worn throughout five 8-day monitoring periods, including the visit day. Activity tracker data were analyzed according to the time, date, and duration of physical activity reported by participants or their caregivers in the ePRO application.

Participants reported AEs at each treatment visit; these were recorded by investigators using the electronic case report form.

QoL was captured using the J-KIDSCREEN-52, the daily-life questionnaires, and the IPAQ by participants. Caregiver comments were collected using survey-based questionnaires on the daily life of participants and caregivers. QoL data collection began from week 5, when participants started on maintenance dosing with emicizumab, with the next data point at week 25, then continuing at 24-week intervals.

These data were collected across different age categories, depending upon the participants' ability to self-report using the ePRO or answer the daily-life questionnaire. Further details on data collection have been previously published. 20

### Data Analysis

All participants included in the data analysis were under 18 years of age. Bleeds, safety, and QoL endpoints were analyzed in all participants who received ≥1 dose of emicizumab after enrolment.

Treated bleeds were events where a coagulation factor product was administered to treat any signs or symptoms of bleeding. An event was considered a single bleed if all further symptoms at the location occurred within 72 hours of the most recent treatment for the bleed. If a bleed occurred more than 72 hours after the last treatment, or in a different location, then it was considered a separate bleed. 24

Physical activities were categorized by risk (low, moderate, and high), with reference to the National Bleeding Disorders Foundation. 25 IPAQ data were categorized by intensity: vigorous, moderate, and light physical activity. 21 Activity intensity was defined using metabolic equivalents of task (METs).

The QoL analysis included all participants, and their caregivers, who responded at least once to the J-KIDSCREEN-52 or the questionnaire-based survey on daily life. For participants who were unable to enter data themselves, their caregivers assisted with completing questionnaires based on participants' responses.

Numerical scores calculated from J-KIDSCREEN-52 were converted to standardized T-scores based on the international average. A mean T-score of 50 denotes an outcome in line with the international average for a specific domain; a mean score of 40 to 60 is within 1 standard deviation (SD) of the international average. 22

Continuous data are presented using means, SDs, medians, first and third quartiles (Q1–Q3), and ranges. Categorical data used frequencies and proportions. Mean and median ABRs for age groups were calculated for treated bleeds using number of bleeds/number of treatment days × 365.25.

All data analyses were descriptive. Owing to the exploratory nature of this analysis, no formal statistical analyses were performed and no intergroup comparison or comparison with a control group was conducted. Missing data were not imputed.

## Results

### Participant Characteristics

Between 1 November 2019 and 31 October 2021, 46 paediatric participants from 25 participating centres enrolled in TSUBASA and received emicizumab. The median (range) age was 3.5 (0–17) years. Most (84.8%) participants had severe HA, and 6 (13.0%) were previously untreated. Following the initial loading dose, most (63.0%) participants received emicizumab at a maintenance dose of 3 mg/kg Q2W ( Table 1 ).

**Table 1:** Baseline characteristics of paediatric participants

	*N* = 46
**Sex,** ***n*** **(%)** Male	46 (100)
**Age** Mean (SD) Median (Q1–Q3) Range	5.5 (5.4) 3.5 (0.0–10.0) 0–17
**Age,** ***n*** **(%)** <2 years ≥2– < 6 years ≥6– < 12 years ≥12– < 18 years	17 (37.0) 8 (17.4) 12 (26.1) 9 (19.6)
**Haemophilia severity,** ***n*** **(%)** Severe Moderate	39 (84.8) 7 (15.2)
**History of haemorrhage** Age <2 years ( *n* = 17): haemorrhage within 12 weeks before administration, *n* (%) None Yes Number of bleeding episodes Mean (SD) Median (Q1–Q3) Range Unknown Age ≥2 years ( *n* = 29): haemorrhage within 24 weeks before administration, *n* (%) None Yes Number of bleeding episodes Mean (SD) Median (Q1–Q3) Range Unknown	4 (23.5) 13 (76.5) *n* = 10 2.5 (2.2) 1.5 (1.0–3.0) 1–8 0 (0.0) 10 (34.5) 17 (58.6) *n* = 9 2.7 (1.5) 2.0 (2.0–3.0) 1–5 2 (6.9)
**Prior use of coagulation factor products,** ***n*** **(%)** None Yes Type of most recent factor replacement therapy Prophylaxis On-demand treatment Unknown	6 (13.0) 40 (87.0) 28 (70.0) 12 (30.0) 0 (0.0)
**Prior treatment with immune tolerance induction therapy,** ***n*** **(%)** None Yes Unknown	41 (89.1) 4 (8.7) 1 (2.2)
**Emicizumab dosing schedule,** ***n*** **(%)** 1.5 mg/kg QW 3 mg/kg Q2W 6 mg/kg Q4W Not classifiable	2 (4.3) 29 (63.0) 8 (17.4) 7 (15.3)
**Presence of target joint,** ***n*** **(%)** None Yes Target joint site Wrist Knee	44 (95.7) 2 (4.3) 1 (2.2) 1 (2.2)

Abbreviations: Q, quartile; Q2W, every 2 weeks; Q4W, every 4 weeks; QW, weekly; SD, standard deviation.

Notes: Target joints were defined as joints with ≥3 bleeds during the 24 weeks prior to study enrolment. Target joints were not defined for participants aged <2 years with no bleeding events in the 24 weeks prior to study enrolment.

### Efficacy

#### ABR and Zero Bleeds

Over a median (Q1–Q3) observation period of 674 (665–690) days, mean (SD) ABR for treated bleeds while receiving emicizumab among paediatric participants was 0.86 (1.26); median (range) ABR for treated bleeds was 0.53 (0.0–5.2). Across the total observation period of 97 weeks, 21 paediatric participants (45.7%) had zero treated bleeds ( Table 2 ). When analyzed in 24-week intervals, the proportion of participants who experienced zero bleeds increased after the initial 24 weeks and remained stable thereafter: baseline to week 24, 63.0%; week 24 to week 48, 82.6%; week 48 to week 72, 78.3%; week 73 to week 96, 76.1% ( Table 3 ). Bleeding rates were low across all age categories ( Table 2 ).

**Table 2:** ABR for treated bleeds and proportion of participants with zero treated bleeds across age categories

	<18 years ( *N* = 46)	<2 years ( *n* = 17)	≥2– < 6 years ( *n* = 8)	≥6– < 12 years ( *n* = 12)	≥12– < 18 years ( *n* = 9)
**ABR**					
Mean (SD)	0.86 (1.26)	0.45 (0.56)	1.40 (1.74)	1.13 (1.38)	0.79 (1.27)
Median (Q1–Q3)	0.53 (0.0–1.1)	0 (0.0–0.54)	0.54 (0.0–2.67)	0.53 (0.0–1.12)	0 (0.0–0.54)
Range	0.0–5.2	0.0–1.7	0.0–5.2	0.0–5.0	0.0–3.9
**Proportion of participants with zero treated bleeds,** ***n*** **(%)**	21 (45.7)	9 (52.9)	3 (37.5)	4 (33.3)	5 (55.6)

Abbreviations: ABR, annualized bleed rate; Q, quartile; SD, standard deviation.

Note: ABRs were calculated using the following formula: number of bleeds/number of days on treatment × 365.25.

**Table 3:** Proportions of participants with treated zero bleeds among those aged <2 years old ( *n* = 17) and <18 years old ( *n* = 46)

	Whole duration of study, *n* (%)	Weeks 1–24, *n* (%)	Weeks 25–48, *n* (%)	Weeks 49–72, *n* (%)	Weeks 73–96, *n* (%)
Overall ( *N* = 129) 19					
0 bleeds	51 (39.5)	74 (57.4)	88 (68.8)	86 (68.3)	92 (73.0)
1–3 bleeds	44 (34.1)	44 (34.1)	32 (25.0)	33 (26.2)	23 (18.3)
4–10 bleeds	25 (19.4)	9 (7.0)	7 (5.5)	6 (4.8)	10 (7.9)
> 10 bleeds	9 (7.0)	2 (1.6)	1 (0.8)	1 (0.8)	1 (0.8)
Aged <2 years ( *n* = 17)					
0 bleeds	9 (52.9)	10 (58.8)	16 (94.1)	14 (82.4)	15 (88.2)
1–3 bleeds	8 (47.1)	7 (41.2)	1 (5.9)	3 (17.6)	2 (11.8)
4–10 bleeds	0 (0.0)	0 (0.0)	0 (0.0)	0 (0.0)	0 (0.0)
> 10 bleeds	0 (0.0)	0 (0.0)	0 (0.0)	0 (0.0)	0 (0.0)
Aged <18 years ( *n* = 46)					
0 bleeds	21 (45.7)	29 (63.0)	38 (82.6)	36 (78.3)	35 (76.1)
1–3 bleeds	17 (37.0)	17 (37.0)	8 (17.4)	9 (19.6)	10 (21.7)
4–10 bleeds	8 (17.4)	0 (0.0)	0 (0.0)	1 (2.2)	1 (2.2)
> 10 bleeds	0 (0.0)	0 (0.0)	0 (0.0)	0 (0.0)	0 (0.0)

#### Physical Activity and Bleeding

Overall, 19/21 participants (aged 6– < 18 years) recorded 172 instances of physical activity during the monitoring periods ( Table 4 ). Of these, 42 were considered low-, 70 moderate-, and 44 high-risk types of activity, with reference to the National Bleeding Disorders Foundation risk classification 25 (16 undefined). Two participants in this age range did not report any instances of physical activity during the monitoring periods. The mean duration of an activity was 62.6 minutes (SD: 67.8) and median duration was 45 minutes (range: 1–477). Median (Q1–Q3) activity intensity was higher for moderate- and high-risk activities (3.67 [2.56–4.92] and 3.56 [1.75–4.33] mean METs, respectively) compared with low-risk activities (1.83 [1.30–2.82] mean METs; Table 4 ). Median (Q1–Q3) activity intensity was also higher for the seven participants aged 12 to <18 years (3.51 [1.43–4.55] mean METs) compared with the 12 participants aged 6 to <12 years (2.97 [1.85–3.92] mean METs; Supplementary Table S1 ). One instance of basketball, a moderate-risk activity, was associated with a traumatic bleed from impact to the head with the basketball. There was no association between traumatic bleeding events and METs.

**Table 4:** Instances of physical activity categorized by risk according to the National Bleeding Disorders Foundation 38

Activity risk categorization ( *n* = number of participants)	Number of instances	Median (Q1–Q3) activity time, minutes	Median (Q1–Q3) activity intensity, mean METs	Median (Q1–Q3) activity intensity, maximum METs
Overall ( *n* = 19)	172	45.0 (19.5–60.0)	3.15 (1.77–4.22)	5.99 (3.60–9.16)
Low risk ( *n* = 12) Walking Golf Swimming Radio calisthenics Fishing Trekking (climbing) Hiking Weight training	42 17 13 6 2 1 1 1 1	44.5 (25.0–100.0)	1.83 (1.30–2.82)	4.01 (2.61–5.73)
Moderate risk ( *n* = 12) Cycling and bicycling Tennis Basketball Dodgeball Rubber-ball baseball Table tennis Jumping rope Softball Jogging/running Soft tennis	70 23 16 7 7 4 4 3 3 2 1	47.0 (15.0–119.0)	3.67 (2.56–4.92)	7.47 (4.38–10.24)
High risk ( *n* = 9) Soccer Athletics and marathon Trampoline Badminton Volleyball Handball Kendo Football Judo	44 13 8 8 5 4 2 2 1 1	45.0 (15.0–50.0)	3.56 (1.75–4.33)	7.79 (3.05–9.46)

Abbreviations: MET, metabolic equivalent of task; Q, quartile.

Note: A total of 16 instances of physical activity were not on the list of risk categorization. MET is the ratio of working metabolic rate relative to resting metabolic rate. One MET is equal to the energy expended when at rest.

#### IPAQ

**Fig. 1:**
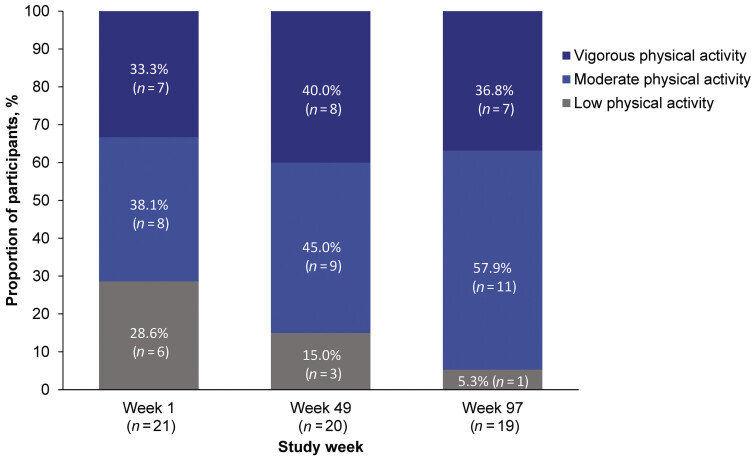
International Physical Activity Questionnaire (IPAQ) scores over the study period. Patients considered unclassifiable for physical activity were excluded.

Over the course of the study, the percentage of participants performing low-intensity physical activity decreased from 28.6% at week 1 to 5.3% at week 97. Additionally, the percentage of participants engaging in moderate-intensity physical activity increased from 38.1% at week 1 to 57.9% at week 97. Percentages for vigorous-intensity physical activity remained similar over the course of the study ( Fig. 1 ). At week 1 ( *n* = 11), participants reported a mean (SD) of 3,106.9 (2,850.6) METs*minutes/week of vigorous activity, which decreased to 1,466.7 (1,447.0) METs*minutes/week at week 97 ( *n* = 12). At week 1 ( *n* = 14), participants reported a mean (SD) of 1,542.9 (1,719.0) METs*minutes/week of moderate activity, which decreased to 1,033.8 (737.4) METs*minutes/week at week 97 ( *n* = 13). At week 1 ( *n* = 18), participants reported a mean (SD) of 1,250.3 (1,224.6) METs*minutes/week of light activity, which increased to 1,438.4 (1,158.7) METs*minutes/week at week 97 ( *n* = 17). In total, paediatric participants who completed the IPAQ at week 1 ( *n* = 21) had a mean (SD) total physical activity of 3,727.7 (4,966.9) METs*minutes/week; at week 97 ( *n* = 19) this was 2,920.7 (2,375.6) METs*minutes/week ( Supplementary Table S2 ).

### Safety

Overall, 26/46 (56.5%) participants <18 years old in TSUBASA experienced a total of 66 AEs, of which two were deemed emicizumab-related: two injection-site reactions in one participant. Four participants experienced one SAE each during the study: COVID-19 infection, anaemia, humerus fracture, and Kawasaki disease. No SAEs were considered emicizumab-related. In 11/46 (23.9%) participants who were tested, none had a positive FVIII inhibitor test during the study. No safety concerns were observed in the six previously untreated participants, and they did not experience any emicizumab-related AEs. No intracranial haemorrhage (ICH) or other safety concerns were noted for participants aged <2 years. No thromboembolic events occurred. All paediatric participants continued to receive emicizumab until study end.

### QoL

J-KIDSCREEN-52 scores were maintained from baseline onwards. All domains showed a trend towards better QoL for adolescents (participants aged 12 to < 16 years) compared with younger children (aged 6 to < 12 years; Supplementary Table S3A ).

Subgroup comparisons of participants who received previous prophylactic or on-demand treatment, participants with moderate or severe HA, and participants with or without bleeds at enrolment were also performed ( Supplementary Table S3B–D ). There were no notable differences except higher mean scores (indicating better QoL) for the ‘school environment’ domain for participants previously receiving prophylaxis versus previously receiving on-demand treatment, at both week 1 and week 97.

Following conversion to mean standardized T-scores, J-KIDSCREEN-52 scores for ‘physical well-being’ (50.6 [SD: 10.6]), ‘self-perception’ (51.1 [7.5]), ‘autonomy’ (53.0 [8.5]), and ‘social support and peers’ (54.8 [12.3]) were close to the international average 22 ( Supplementary Table S4 ). For the ‘school environment’ domain, mean (SD) T-scores were relatively stable between week 1 (50.8 [15.1]) and week 73 (51.8 [13.9]). At week 97, the mean (SD) T-score was 59.0 (11.3), which is close to ± 1 SD of the international average.

#### Questionnaire on Daily Life in Participants Aged 6 to <18 Years

**Fig. 2:**
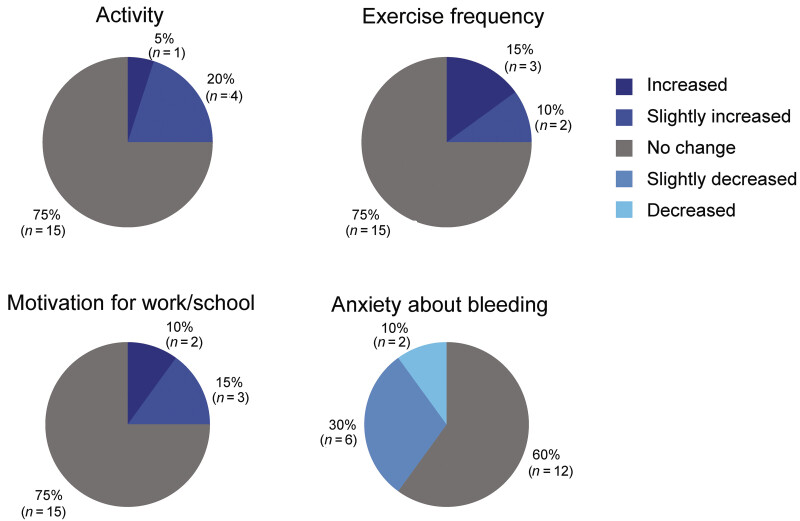
Questionnaire answered by participants on their daily life: changes at week 97 compared with pre-emicizumab treatment ( *n* = 20). For participants who were unable to enter data themselves, the caregiver of the participant assisted with completing the questionnaire based on the responses of the participant.

At week 1, 9/21 (42.9%) participants reported that they may be restricted in their exercise, 7/21 (33.3%) participants reported that they may be limited in their activities, and 4/21 (19.0%) participants reported limiting their participation in physical education depending on the type of activity. At week 97, of the 20 participants who completed the questionnaire, 5 (25%) participants reported increased/slightly increased frequency of physical activity, and 5 (25%) participants reported increased/slightly increased activity, suggesting an activity-related QoL improvement ( Fig. 2 ; the remainder reported no change). Five (25%) participants reported increased or slightly increased motivation for work/school and other initiatives, and eight (40%) participants reported decreased or slightly decreased anxiety about bleeding (for both measures, the remainder reported no change).

#### Questionnaire on Daily Life in Caregivers

**Fig. 3:**
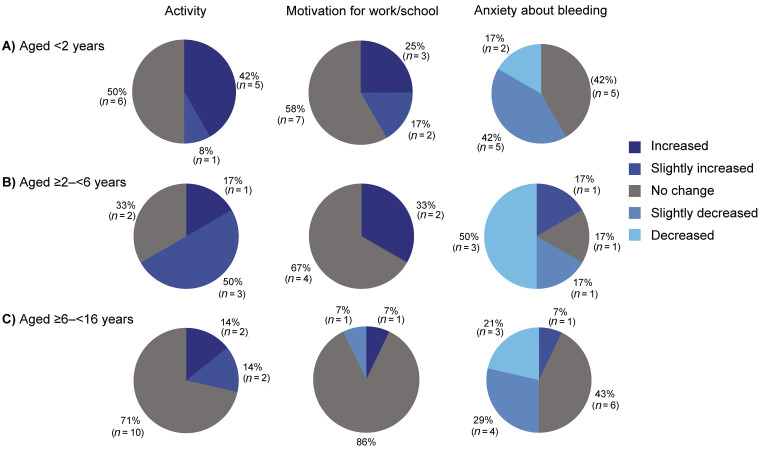
Questionnaire answered by caregivers on the daily life of their children: changes at week 97 compared with pre-emicizumab by age category of participant ( *n* = 32). (A) Aged <2 years. (B) Aged ≥2 to <6. (C) Aged ≥6 to <16. Note: Percentages may not add up to 100% due to rounding.

At week 1, 39 caregivers of paediatric participants aged up to 12 years completed the questionnaire, including providing comments ( Supplementary Table S5 ), with around a third reporting limitations and restrictions in activity and school life, and most (87.2%) reporting that they were concerned about bleeding in their child. At week 97, 32 caregivers completed the questionnaire ( Fig. 3 ). In all 14 (43.8%) caregivers reported that their child's activity had increased/slightly increased, while 18 (56.3%) caregivers reported no change. Eight (25.0%) caregivers reported their child's motivation for school and other initiatives had increased/slightly increased, while 23 (71.9%) reported no change. Around half (18 caregivers, 56.3%) reported that their anxiety about their child's bleeding had decreased/slightly decreased, while 12 (37.5%) reported no change.

## Discussion

Children and adolescents who participated in TSUBASA ( *n* = 46) experienced low ABRs with no unexpected safety signals. Only one participant had a bleed associated with physical activity (basketball). Compared with week 1, a greater proportion of participants reported moderate-intensity physical activity at week 97, and a smaller proportion reported low-intensity activity. QoL was maintained according to J-KIDSCREEN-52. A daily-life questionnaire for participants and their caregivers revealed improvements in activity and motivation for school, and reduced anxiety about bleeding, in a reasonable proportion of children and adolescents who completed the questionnaire. Almost all participants who did not report improvement maintained their baseline responses.

Results of TSUBASA are in line with previous clinical trials and real-world data, supporting the use of emicizumab prophylaxis in this population. 9 12 13 14 26 27 28 Across HAVEN 2 (children with HA with FVIII inhibitors aged <12 years), HOHOEMI (Japanese children with HA without FVIII inhibitors aged 4 months–10 years), and HAVEN 7 (infants with HA without FVIII inhibitors aged ≤12 months), model-based ABRs for treated bleeds were low (ranging from 0.2 to 1.3) 9 12 13 ; the mean (0.86) and median ABR (0.53) for treated bleeds observed in TSUBASA fell within this expected range. Initiating emicizumab prophylaxis early in life may confer joint health benefits, although further research is required to evaluate this and any successive gains in physical activity. The 7-year follow-up of HAVEN 7 will provide further data on this topic. 12 Safety outcomes in TSUBASA were also consistent with previous clinical trial findings. As seen in HAVEN 7, 12 no instances of ICH were reported, despite 37% of participants being <2 years of age at study initiation. Although not a study endpoint, these data could support the theory that emicizumab prophylaxis may reduce ICH risk, which is still an area of concern in infants with haemophilia. 29

A study conducted in 2020 was designed to compare health-related QoL in Japanese children and adolescents with haemophilia (94.4% of whom were receiving prophylaxis), with that of a control group, using J-KIDSCREEN-52. 30 Compared with healthy controls, children with haemophilia (aged 8–12 years) reported significantly lower scores for ‘moods and emotions’; however, those aged 13 to 18 years reported similar scores in all self-assessed QoL domains. These reports align with the TSUBASA results, as all domains of J-KIDSCREEN-52 indicated better QoL for adolescents (aged 12– < 16 years), compared with younger children (aged 6– < 12 years). Differences in QoL scores between younger and older participants could potentially be explained by a larger influence of caregivers on the reporting of data for younger children and overestimation of disease burden, although the certainty of this could not be established. J-KIDSCREEN-52 scores were maintained close to the international average throughout the duration of the TSUBASA study. 22

In TSUBASA, adolescents aged 12 to <18 years engaged in more intense physical activity (median [Q1–Q3] activity intensity of 3.51 [1.43–4.55] mean METs, indicating ‘moderate’ activity) 31 than children aged 6 to <12 years (median [Q1–Q3] activity intensity of 2.97 [1.85–3.92] mean METs, indicating ‘light’ activity). 31 This was corroborated by the IPAQ data in this study, which saw a 19.8% increase in participants reporting moderate physical activity and a 23.3% decrease in light physical activity by week 97. This difference is perhaps due to school club activities, as the most common activity in the adolescent age group was tennis, with two participants recording 16 instances collectively, whereas the most common activity in the child age group was walking, with six participants recording 14 instances collectively. Even with an average moderate intensity of activity (and some children and adolescents reaching METs that indicate ‘vigorous’ activity), no bleeding occurred during physical activity while receiving emicizumab for adolescents, and only one bleed occurred in a child. This bleed was a traumatic bleed due to the impact of the ball on the child's head, and thus not related to activity intensity. The single incidence of bleeding associated with physical activity should be interpreted with caution, however, as the monitoring period was limited to five 8-day observation windows. This may have resulted in underestimation of the incidence of activity-related bleeding. Longer and more continuous observation in future studies may provide a more accurate insight into the association between physical activity and bleeding risk. Overall, 2/21 participants (aged 6– < 18 years) did not record any instances of physical activity. This may be due to several reasons, including simply that they did not do any activity within the monitoring periods, failure to remember to report using the ePRO mobile application, the burden and complexity of reporting physical activity events, and technical barriers such as software glitches.

Although no other studies have specifically investigated the relationship between physical activity and bleeding in children and adolescents with HA receiving emicizumab prophylaxis, similar studies have been conducted in those receiving factor prophylaxis. In a retrospective analysis of 37 children and adolescents (aged 5–20 years) with severe haemophilia, who were receiving factor prophylaxis and partaking in high- and low-impact activities, significant bleeding complications were uncommon. 32 A study of 15 children and adolescents (aged 5–18 years) with haemophilia (66.7% severe; 73.3% receiving prophylaxis) found no increase in bleeding events over the study period, during which participants increased their physical activity and participated in sports and fitness, compared with the previous year. 33 Notably, the authors encouraged participation in physical activity and there was no increased risk for high-impact activities versus low-impact activities. 32 In contrast, a later case-crossover study nested within a prospective cohort study, which included 104 children and adolescents aged 4 to 18 years with moderate or severe haemophilia (85.6% receiving prophylaxis), reported that ‘vigorous’ physical activity was transiently associated with a moderate risk of increased bleeding. 34 Nevertheless, the absolute risk increase of bleeding associated with physical activity was thought to be small. 34 No conclusions can be drawn on the likelihood of bleeding with high- versus low-risk activities in TSUBASA, due to only one activity-associated bleed occurring in participants aged <18 years. The results of TSUBASA are concordant with the limited literature on the relationship between physical activity and bleeding in children and adolescents with HA, indicating that for those receiving prophylaxis, any increased risk of bleeding with physical activity is minimal. These findings provide further encouragement for children and adolescents with HA receiving prophylactic treatment, such as emicizumab, to participate in physical activity.

### Study Limitations

Owing to the limited sample size in this study, only descriptive comparisons are provided; no formal statistical hypothesis testing was performed. Future studies with larger sample sizes are required for verification of these results. The number of participants reporting their exercise was limited by the age distribution of the paediatric population in this study, as 41.3% of paediatric participants were <6 years old. Types of physical activity, activity duration, and bleeding time were patient-reported outcomes, which are subject to participant interpretation and may not be rigorously recorded. Activity intensity (captured using a wearable activity tracker) was analyzed with reference to patient-reported duration of the activity, thereby restricting its accuracy (e.g., if the participant did not correctly record the time and duration of the activity). Although all treated bleeding events were documented throughout the study (including bleeds associated with physical activity), physical activity data were only recorded during defined 8-day periods, meaning activity status during the remainder of the study was unknown. This is a notable limitation of the study, as it is possible that some rare but clinically significant activity-related bleeding events may not have been captured.

With regard to limitations of the daily-life questionnaire, this had a target sample size of 20 participants and was completed by 20 participants at week 97; the small number completing this questionnaire may introduce some bias. Second, it was not investigated if any change in activity post-emicizumab initiation reported by the questionnaire was corroborated by activity tracker data, as the activity tracker was not worn prior to receiving emicizumab treatment. Third, participants who were able to complete the questionnaire at 97 weeks had also successfully received emicizumab prophylaxis for this duration, and therefore may give a more positive account of their treatment experience. No paediatric participants discontinued emicizumab treatment before 97 weeks. Finally, the COVID-19 pandemic may have impacted QoL of study participants, particularly regarding physical activity and school attendance, as has been reported in other studies. 35 36 37 According to the results of the questionnaire on daily life, percentage of missed class time was higher at later assessments than at week 1; although the questionnaire asked about time missed due to haemophilia, it may have been difficult for participants to accurately distinguish between this and time missed due to the pandemic. Reasons for missed class time were not collected, so any conclusions drawn from these data would be speculative.

## Conclusion

These results from the TSUBASA study suggest that children and adolescents with HA receiving regular emicizumab prophylaxis are able to engage in consistent levels of various physical activities, with low risk of associated bleeds. QoL appeared to be maintained for 2 years after starting emicizumab treatment. Emicizumab was well tolerated, with no occurrence of ICH, a common problem in infants with HA, or thromboembolic events. No new safety signals were observed.

## Notes

**What is Known About This Topic?**

- Emicizumab is an established prophylactic treatment shown to reduce bleeding episodes in people with haemophilia A.
- The efficacy and safety of emicizumab have been demonstrated primarily in the adult population and mixed paediatric/adult studies.
- Maintaining physical activity and quality of life are key management goals for paediatric patients with haemophilia.

**What Does This Paper Add?**

- This paper provides specific data on physical activity, bleeding outcomes, and quality of life in a Japanese paediatric cohort with haemophilia A receiving emicizumab.
- It presents a subanalysis of the TSUBASA study, offering focused evidence on the paediatric population.
- The findings demonstrate the safety and effectiveness of emicizumab in paediatric patients, contributing to improved physical activity and quality of life, which supports its use in this specific patient group.
