# Supplementary material for: Paediatric Subanalysis of TSUBASA, Assessing Physical Activity, Bleeding, Quality of Life and Safety in People with Haemophilia A Receiving Emicizumab
Source: TH Open. 2026 Jan 21;10:a27818278. doi: 10.1055/a-2781-8278 (PMC12828975; doi:10.1055/a-2781-8278)
Supplement: Supplementary file 1 — Supplementary Material [file 10-1055-a-2781-8278_27946179.pdf]

SUPPLEMENTARY MATERIAL

Paediatric Subanalysis of TSUBASA, Assessing Physical Activity, Bleeding, Quality of Life and Safety in People with Haemophilia A Receiving Emicizumab

Keiji Nogami, Kagehiro Amano, Akihiro Sawada, Azusa Nagao,[5] Chiai Nagae, Masanori Nojima, Nobuaki Suzuki, Mika Kawano, Tomomi Shimura, Yoshimasa Sugao, Teruhisa Fujii

Supplementary Results

Incidence of activity-associated bleed in one participant

One participant with severe haemophilia A and aged 9 years experienced an activity-associated anterior parietal bleed while playing basketball due to impact of the basketball with their head. The bleed occurred after 20 minutes of activity time; the mean metabolic equivalent of task (MET) was 4.20 and the maximum MET was 9.92. The participant was observed to have a swelling of 2–3 cm and received rurioctocog alfa. The bleed was recorded at the Week-49 visit.

TH Open | DOI 10.1055/a-2781-8278 | © The Author(s).

Supplementary Table S1. Physical activity status among participants aged 6–<12 years (n=12) and 12–18 years (n=9)

Activity risk (n=number of participants)	Number of instances	Median (Q1–Q3) activity time, minutes	Median (Q1–Q3) activity intensity, mean METs	Median (Q1–Q3) activity intensity, maximum METs
**Participants aged 6–<12 years**				
Overall (n=12)	97	30.0 (15.0–60.0)	2.97 (1.85–3.92)	5.85 (4.05–8.92)
Low risk (n=11)	23	60.0 (30.0–88.0)	2.17 (1.28–3.43)	4.93 (3.10–7.83)
Moderate risk (n=7)	38	27.5 (10.0–55.0)	3.17 (2.31–3.76)	5.55 (4.26–8.09)
High risk (n=5)	22	27.5 (10.0–45.0)	3.56 (1.80–4.65)	7.82 (4.39–9.35)
**Participants aged 12–18 years**				
Overall (n=7)*	75	50.0 (30.0–120.0)	3.51 (1.43–4.55)	6.80 (3.30–10.24)
Low risk (n=4)	19	40.0 (25.0–135.0)	1.47 (1.30–2.20)	3.37 (1.66–4.95)
Moderate risk (n=5)	32	60.0	4.40	9.03

TH Open | DOI 10.1055/a-2781-8278 | © The Author(s).

		(49.5–128.5)	(3.57–5.38)	(7.81–11.87)
High risk (n=4)	22	50.0<br><br>(45.0–50.0)	3.61<br><br>(1.28–4.21)	7.73<br><br>(1.34–10.47)

*Two participants aged 12–18 years old did not report any physical activity during the five 8-day monitoring periods. MET is equal to the energy expended when at rest. Sixteen activities listed do not have a risk category.
MET, metabolic equivalent of task; Q, quartile.

TH Open | DOI 10.1055/a-2781-8278 | © The Author(s).

Supplementary Table S2. Changes in responses to the IPAQ for participants aged ≥6 years (n=21) from Week 1 to Week 97

IPAQ Domain	Week 1	Week 49		Week 97	
	Evaluation value	Evaluation value	Change from Week 1	Evaluation value	Change from Week 1
**Amount of vigorous physical activity METs*mins/week**					
Number of participants	11	11	7	12	6
Mean (SD)	3106.9 (2850.6)	2640.0 (2960.9)	-13.7 (1086.4)	1466.7 (1447.0)	-1400.0 (2154.2)
Q1–Q3	960.0–7200.0	480.0–6720.0	-480.0–0.0	440.0–2640.0	-3600.0–-480.00
Median	1296.0	960.0	-480.0	880.0	-720.0
Range	960.0–7200.0	0.0–7200.0	-720.0–2400.0	80.0–4320.0	-4320.0–1440.0
**Amount of moderate physical activity METs*mins/week**					
Number of participants	14	13	10	13	8
Mean (SD)	1542.9 (1719.0)	827.7 (915.4)	-840.0 (1294.6)	1033.8 (737.4)	-490.0 (1542.8)
Q1–Q3	360.0–2400.0	240.0–1200.0	-1680.0–240.0	480.0–1440.0	-1320.0–500.0
Median	760.0	480.0	-420.0	960.0	120.0

TH Open | DOI 10.1055/a-2781-8278 | © The Author(s).

Range	240.0–5040.0	120.0–3360.0	-3360.0–400.0	120.0–2400.0	-3240.0–720.0
**Amount of light physical activity METs*mins/week**					
Number of participants	18	18	15	17	16
Mean (SD)	1250.3 (1224.6)	1427.3 (1287.7)	337.7 (1521.4)	1438.4 (1158.7)	315.6 (1901.3)
Q1–Q3	396.0–1386.0	412.5–2376.0	-396.0–1023.0	594.0–1485.0	-610.5–1658.3
Median	825.0	907.5	165.0	1188.0	305.3
Range	132.0–4158.0	165.0–4158.0	-2524.5–3762.0	247.5–4158.0	-2772.0–3432.0
**Total physical activity METs*mins/week**					
Number of participants	21	18	18	19	19
Mean (SD)	3727.7 (4966.9)	3638.4 (3810.0)	460.1 (3495.3)	2920.7 (2375.6)	-357.2 (4274.6)
Q1–Q3	720.0–2865.0	967.5–4755.0	-877.5–1812.0	800.0–4890.0	-576.0–1812.0
Median	1398.0	1717.5	234.8	2346.0	3.0
Range	247.5–16398.0	660.0–12456.0	-6604.5–8083.5	367.5–7656.0	-9012.0–7408.5

Physical activity data where the total daily physical activity time exceeds 960 minutes (16 hours) were excluded from tabulation (sleep time was estimated to be 8 hours). Data where the number of days of physical activity exceeded 7 days for each type of physical activity were excluded from tabulation. If daily activity time for a physical activity is <10 minutes, the number of activity days and activity time will be replaced with zero. Any activity time >180 minutes (3 hours) will be coded as ’180 minutes’. Light physical activity includes activities such as walking.

TH Open | DOI 10.1055/a-2781-8278 | © The Author(s).

IPAQ, International Physical Activity Questionnaire; METs, metabolic equivalent of task; mins, minutes; Q, quartile; SD, standard deviation.

TH Open | DOI 10.1055/a-2781-8278 | © The Author(s).

Supplementary Table S3A. J-KIDSCREEN-52 QoL results receiving prophylaxis (n=16) and on-demand treatment (n=3) split by age group at enrolment

	**Age category**														
Age group	**Total population (≥6−<18 years)**					**≥6−<12 years**					**≥12−<16 years**				
Time	Wk 1	Wk 25	Wk 49	Wk 73	Wk 97	Wk 1	Wk 25	Wk 49	Wk 73	Wk 97	Wk 1	Wk 25	Wk 49	Wk 73	Wk 97
**Number of participants (n)**	19	19	18	16	18	12	12	11	9	11	7	7	7	7	7
**Physical wellbeing**															
Mean	19.2	19.6	19.2	19.1	19.7	19.2	18.3	18.5	17.7	19.0	19.3	21.9	20.3	21.0	20.7
(SD)	(3.6)	(3.2)	(3.9)	(3.9)	(4.1)	(2.7)	(2.9)	(4.0)	(3.3)	(4.6)	(5.1)	(2.2)	(3.7)	(4.0)	(3.2)
Range	10–25	14–25	13–25	13–25	8–25	15–24	14–25	13–25	13–23	8–23	10–25	20–25	15–25	13–25	15–25
**Psychological wellbeing**															
Mean	25.7	26.1	25.1	24.2	26.4	24.3	24.7	23.5	23.2	25.5	28.0	28.6	27.4	25.4	27.9
(SD)	(4.2)	(3.7)	(4.2)	(4.4)	(3.8)	(3.6)	(3.4)	(4.5)	(4.4)	(4.4)	(4.4)	(2.9)	(2.4)	(4.4)	(2.1)
Range	18–30	19–30	18–30	15–30	20–30	18–30	19–30	18–30	15–30	20–30	18–30	22–30	23–30	18–30	24–30
**Moods & emotions**															

TH Open | DOI 10.1055/a-2781-8278 | © The Author(s).

Mean	29.9	31.2	29.8	28.8	30.7	28.3	29.8	27.8	27.3	30.5	32.6	33.6	33.0	30.6	30.9
(SD)	(4.4)	(3.9)	(3.7)	(4.5)	(4.0)	(4.3)	(4.2)	(3.2)	(3.4)	(3.5)	(3.1)	(1.5)	(1.9)	(5.3)	(5.0)
Range	23–35	22–35	22–35	20–35	21–35	23–35	22–35	22–35	20–32	24–35	26–35	31–35	30–35	23–35	21–35
**Self-perception**															
Mean	20.9	20.2	20.2	19.3	20.7	19.7	18.8	19.6	18.7	19.8	23.0	22.6	21.1	20.1	22.1
(SD)	(2.7)	(3.5)	(3.1)	(3.1)	(2.7)	(2.0)	(3.3)	(2.6)	(2.9)	(2.8)	(2.5)	(2.5)	(3.7)	(3.4)	(2.2)
Range	16–25	13–25	14–25	14–25	15–25	16–24	13–23	15–25	14–22	15–24	19–25	18–25	14–25	16–25	20–25
**Autonomy**															
Mean	21.6	21.1	20.4	20.4	21.0	20.7	20.4	18.9	18.8	20.3	23.3	22.1	22.9	22.4	22.1
(SD)	(2.5)	(2.5)	(3.4)	(3.6)	(3.0)	(2.0)	(2.5)	(2.7)	(3.4)	(2.5)	(2.4)	(2.3)	(3.0)	(2.7)	(3.5)
Range	18–25	17–25	14–25	14–25	15–25	18–25	17–25	14–25	14–25	17–25	19–25	19–25	18–25	19–25	15–25
**Parent relation & home life**															
Mean	26.2	25.6	25.4	24.9	25.3	24.4	23.8	23.7	23.6	24.0	29.1	28.7	28.0	26.6	27.3
(SD)	(4.7)	(4.2)	(4.1)	(4.6)	(4.3)	(5.0)	(4.0)	(4.0)	(4.6)	(4.4)	(2.3)	(2.2)	(2.8)	(4.2)	(3.5)
Range	16–30	17–30	18–30	16–30	18–30	16–30	17–30	18–30	16–30	18–30	24–30	25–30	23–30	20–30	20–30
**Social support & peers**															

TH Open | DOI 10.1055/a-2781-8278 | © The Author(s).

Mean	22.8	24.9	23.7	23.4	25.2	20.9	23.1	21.8	20.0	24.2	26.0	28.1	26.6	27.7	26.7
(SD)	(6.9)	(4.2)	(4.8)	(6.0)	(4.5)	(4.9)	(4.1)	(4.0)	(5.9)	(4.7)	(8.9)	(1.5)	(4.7)	(2.3)	(4.1)
Range	6–30	17–30	15–30	6–30	15–30	13–29	17–29	15–29	6–26	15–30	6–30	26–30	17–30	25–30	19–30
**School environment**															
Mean	22.2	24.0	22.3	23.0	25.7	20.8	22.3	18.5	19.4	24.5	24.4	27.0	28.4	27.6	27.6
(SD)	(6.9)	(4.1)	(7.7)	(6.4)	(4.0)	(5.5)	(3.6)	(7.6)	(6.5)	(4.3)	(8.7)	(3.3)	(1.3)	(1.6)	(2.5)
Range	6–30	14–30	6–30	6–30	19–30	8–29	14–27	6–29	6–28	19–30	6–30	22–30	26–30	25–30	24–30
**Social acceptance (bullying)**															
Mean	13.1	13.2	13.8	13.4	13.3	12.3	12.3	13.2	12.3	12.7	14.6	14.6	14.7	14.9	14.3
(SD)	(2.5)	(2.2)	(1.7)	(2.0)	(1.9)	(2.7)	(2.3)	(1.9)	(2.1)	(2.0)	(1.1)	(1.1)	(0.5)	(0.4)	(1.5)
Range	7–15	8–15	10–15	9–15	10–15	7–15	8–15	10–15	9–15	10–15	12–15	12–15	14–15	14–15	11–15
**Financial resources**															
Mean	9.5	10.3	10.4	9.8	10.9	8.8	9.3	8.2	8.9	9.7	10.7	12.0	13.9	11.0	12.9
(SD)	(4.2)	(3.7)	(4.7)	(3.8)	(4.1)	(4.2)	(3.9)	(4.2)	(2.9)	(4.6)	(4.3)	(3.1)	(3.0)	(4.6)	(2.3)

TH Open | DOI 10.1055/a-2781-8278 | © The Author(s).

Range	3–15	3–15	3–15	3–15	3–15	3–15	3–15	3–15	3–12	3–15	4–15	8–15	7–15	3–15	9–15

The J-KIDSCREEN-52 questionnaire was only completed by participants aged 6 to <16 years old.

QoL, quality of life; SD, standard deviation; Wk, week

TH Open | DOI 10.1055/a-2781-8278 | © The Author(s).

Thieme

Supplementary Table S3B. J-KIDSCREEN-52 QoL results for all participants receiving prophylaxis (n=16) and on-demand treatment (n=3) at enrolment

QoL endpoints	Prophylaxis					On-demand treatment				
	Wk1	Wk25	Wk49	Wk73	Wk97	Wk1	Wk25	Wk49	Wk73	Wk97
**Number of participants (n)**	16	16	15	13	15	3	3	3	3	3
**Physical wellbeing**										
Mean (SD)	19.4 (3.8)	19.7 (3.3)	18.8 (3.7)	19.0 (4.0)	19.8 (4.2)	18.0 (2.6)	19.0 (2.6)	21.0 (5.3)	19.7 (4.2)	19.0 (4.6)
Median	19.5	20.0	19.0	20.0	21.0	19.0	20.0	23.0	21.0	20.0
Q1–Q3	17.0–22.0	17.0–21.0	16.0–22.0	17.0–21.0	18.0–22.0	15.0–20.0	16.0–21.0	15.0–25.0	15.0–23.0	14.0–23.0
Range	10–25	14–25	13–25	13–25	8–25	15–20	16–21	15–25	15–23	14–23
**Psychological wellbeing**										
Mean (SD)	26.2 (3.9)	26.1 (3.4)	25.2 (4.1)	24.6 (4.8)	27.0 (3.7)	23.0 (5.6)	26.0 (6.1)	24.3 (5.7)	22.3 (0.6)	23.3 (3.5)
Median	27.5	26.0	24.0	25.0	29.0	22.0	29.0	26.0	22.0	23.0
Q1–Q3	23.5–30.0	24.0–29.5	21.0–30.0	24.0–28.0	24.0–30.0	18.0–29.0	19.0–30.0	18.0–29.0	22.0–23.0	20.0–27.0
Range	18–30	19–30	18–30	15–30	20–30	18–29	19–30	18–29	22–23	20–27
**Moods & emotions**										
Mean (SD)	30.2 (4.2)	31.3 (3.8)	29.8 (3.8)	28.8 (4.5)	30.9 (3.8)	28.3 (5.8)	30.3 (5.0)	30.0 (4.4)	28.7 (5.5)	29.3 (5.5)
Median	31.5	33.0	30.0	28.0	32.0	25.0	31.0	28.0	26.0	29.0

TH Open | DOI 10.1055/a-2781-8278 | © The Author(s).

Q1–Q3	26.5–33.5	29.0–35.0	28.0–33.0	27.0–32.0	28.0–34.0	25.0–35.0	25.0–35.0	27.0–35.0	25.0–35.0	24.0–35.0
Range	23–35	22–35	22–35	20–35	21–35	25 – 35	25–35	27–35	25–35	24–35
**Self-perception**										
Mean (SD)	21.1 (2.5)	20.5 (3.4)	20.1 (3.2)	19.2 (3.2)	21.0 (2.9)	19.7 (4.0)	18.7 (4.0)	20.7 (3.1)	19.7 (3.2)	19.3 (1.2)
Median	20.0	21.0	20.0	20.0	22.0	19.0	21.0	20.0	21.0	20.0
Q1–Q3	19.5–23.5	18.0–23.0	18.0–22.0	16.0–21.0	19.0–24.0	16.0–24.0	14.0–21.0	18.0–24.0	16.0–22.0	18.0–20.0
Range	18–25	13–25	14–25	14–25	15–25	16–24	14–21	18–24	16–22	18–20
**Autonomy**										
Mean (SD)	21.7 (2.7)	20.9 (2.7)	20.4 (3.4)	20.7 (3.2)	21.1 (3.1)	21.3 (0.6)	21.7 (1.5)	20.7 (3.8)	19.0 (5.6)	20.7 (2.9)
Median	22.0	21.5	20.0	20.0	21.0	21.0	22.0	19.0	18.0	19.0
Q1–Q3	19.0–25.0	19.0–22.0	18.0–24.0	18.0–23.0	19.0–24.0	21.0–22.0	20.0–23.0	18.0–25.0	14.0–25.0	19.0–24.0
Range	18–25	17–25	14–25	16–25	15–25	21–22	20–23	18–25	14–25	19–24
**Parent relation & home life**										
Mean (SD)	26.7 (4.5)	25.6 (4.6)	25.3 (4.2)	24.7 (4.8)	25.1 (4.5)	23.3 (5.9)	25.7 (0.6)	26.0 (4.0)	25.7 (3.8)	26.0 (3.5)
Median	30.0	26.5	24.0	25.0	27.0	21.0	26.0	26.0	24.0	24.0
Q1–Q3	24.0–30.0	21.0–30.0	23.0–30.0	20.0–30.0	20.0–30.0	19.0–30.0	25.0–26.0	22.0–30.0	23.0–30.0	24.0–30.0
Range	16–30	17–30	18–30	16–30	18–30	19–30	25–26	22–30	23–30	24–30
**Social support & peers**										

TH Open | DOI 10.1055/a-2781-8278 | © The Author(s).

Mean (SD)	22.3 (7.3)	24.8 (4.3)	23.9 (4.6)	24.2 (4.2)	25.1 (4.9)	25.3 (4.2)	26.0 (3.5)	22.3 (6.7)	19.7 (11.8)	25.3 (2.5)
Median	23.0	26.0	24.0	24.0	25.0	24.0	28.0	24.0	26.0	25.0
Q1–Q3	17.5–29.0	21.0–28.0	20.0–29.0	21.0–27.0	21.0–30.0	22.0–30.0	22.0–28.0	15.0–28.0	6.0–27.0	23.0–28.0
Range	6–30	17–30	17–30	18–30	15–30	22–30	22–28	15–28	6–27	23–28
**School environment**										
Mean (SD)	22.6 (7.4)	24.9 (3.5)	23.5 (6.9)	23.8 (6.3)	26.2 (3.5)	19.7 (1.5)	19.0 (4.4)	16.7 (11.0)	19.3 (6.5)	23.0 (6.1)
Median	24.5	25.0	26.0	25.0	27.0	20.0	21.0	16.0	19.0	20.0
Q1–Q3	19.0–28.5	23.0–28.0	21.0–29.0	22.0–28.0	23.0–30.0	18.0–21.0	14.0–22.0	6.0–28.0	13.0–26.0	19.0–30.0
Range	6–30	18–30	6–30	6–30	20–30	18–21	14–22	6–28	13–26	19–30
**Social acceptance (bullying)**										
Mean (SD)	13.3 (2.5)	13.8 (1.5)	13.8 (1.7)	14.0 (1.5)	13.4 (2.0)	12.3 (3.1)	9.7 (2.1)	13.7 (2.3)	11.0 (2.6)	13.0 (2.0)
Median	14.5	15.0	15.0	15.0	15.0	13.0	9.0	15.0	10.0	13.0
Q1–Q3	12.0–15.0	13.0–15.0	13.0–15.0	13.0–15.0	12.0–15.0	9.0–15.0	8.0–12.0	11.0–15.0	9.0–14.0	11.0–15.0
Range	7–15	11–15	10–15	11–15	10–15	9–15	8–12	11–15	9–14	11–15
**Financial resources**										
Mean (SD)	10.3 (4.0)	10.6 (3.4)	10.3 (4.5)	10.9 (2.9)	11.5 (3.9)	5.3 (3.2)	8.7 (6.0)	10.7 (6.7)	5.0 (3.5)	8.0 (4.6)
Median	10.0	10.0	9.0	11.0	12.0	4.0	8.0	14.0	3.0	9.0

TH Open | DOI 10.1055/a-2781-8278 | © The Author(s).

Q1–Q3	6.5–14.5	9.0–13.5	6.0–15.0	9.0–12.0	9.0–15.0	3.0–9.0	3.0–15.0	3.0–15.0	3.0–9.0	3.0–12.0
Range	3–15	4–15	3–15	6–15	3–15	3–9	3–15	3–15	3–9	3–12

The J-KIDSCREEN-52 questionnaire was only completed by participants aged 6 to <16 years old.

Q, quartile; QoL, quality of life; SD, standard deviation; Wk, week

TH Open | DOI 10.1055/a-2781-8278 | © The Author(s).

Supplementary Table S3C. J-KIDSCREEN-52 QoL results for all participants with severe (n=14) and moderate (n=5) haemophilia A

QoL endpoints	Severe HA					Moderate HA				
	Wk1	Wk25	Wk49	Wk73	Wk97	Wk1	Wk25	Wk49	Wk73	Wk97
**Number of participants (n)**	14	14	13	12	14	5	5	5	4	4
**Physical wellbeing**										
Mean (SD)	19.5 (4.2)	19.4 (3.6)	18.6 (3.8)	19.1 (4.0)	19.7 (4.4)	18.4 (1.3)	20.2 (1.3)	20.6 (4.3)	19.3 (4.3)	19.5 (3.3)
Median	20.0	19.0	19.0	19.0	21.5	19.0	21.0	23.0	20.5	20.0
Q1–Q3	17.0–23.0	17.0–21.0	16.0–20.0	16.0–21.5	18.0–22.0	17.0–19.0	20.0–21.0	16.0–23.0	16.5–22.0	17.5–21.5
Range	10–25	14–25	13–25	13–25	8–25	17–20	18–21	16–25	13–23	15–23
**Psychological wellbeing**										
Mean (SD)	25.4 (4.3)	25.3 (3.8)	24.6 (4.6)	24.7 (4.5)	26.2 (4.1)	26.6 (4.2)	28.4 (2.5)	26.2 (3.1)	22.8 (4.1)	27.0 (2.9)
Median	25.5	25.0	24.0	24.5	28.0	29.0	29.0	27.0	22.5	27.5
Q1–Q3	23.0–29.0	22.0–29.0	21.0–30.0	23.0–28.5	21.0–30.0	22.0–30.0	29.0–30.0	26.0–28.0	20.0–25.5	25.0–29.0
Range	18–30	19–30	18–30	15–30	20–30	22–30	24–30	21–29	18–28	23–30

TH Open | DOI 10.1055/a-2781-8278 | © The Author(s).

**Moods & emotions**										
Mean (SD)	30.5 (4.1)	30.4 (4.2)	29.6 (3.9)	29.0 (4.6)	31.4 (3.3)	28.2 (5.1)	33.4 (1.7)	30.4 (3.6)	28.0 (4.8)	28.3 (5.7)
Median	32.0	30.0	28.0	28.5	32.5	26.0	33.0	30.0	26.0	28.5
Q1–Q3	27.0–34.0	27.0–35.0	28.0–33.0	27.0–33.0	28.0–34.0	25.0–32.0	33.0–35.0	27.0–33.0	25.0–31.0	24.5–32.0
Range	23–35	22–35	22–35	20–35	24–35	23–35	31–35	27–35	25–35	21–35
**Self-perception**										
Mean (SD)	20.7 (2.7)	19.4 (3.7)	19.9 (3.3)	19.3 (3.4)	20.6 (3.0)	21.4 (2.9)	22.4 (1.7)	21.0 (2.5)	19.3 (2.4)	21.0 (2.0)
Median	20.0	19.0	20.0	20.0	21.0	20.0	22.0	21.0	20.0	20.0
Q1–Q3	19.0–23.0	17.0–23.0	18.0–22.0	16.0–21.5	18.0–23.0	19.0–24.0	21.0–23.0	19.0–23.0	17.5–21.0	20.0–22.0
Range	16–25	13–25	14–25	14–25	15–25	19–25	21–25	18–24	16–21	20–24
**Autonomy**										
Mean (SD)	21.6 (2.6)	21.1 (2.8)	20.3 (3.5)	20.0 (3.7)	21.2 (2.8)	21.8 (2.2)	21.0 (1.6)	20.8 (3.4)	21.5 (3.1)	20.3 (4.1)
Median	21.5	22.0	20.0	19.5	20.5	22.0	21.0	19.0	21.5	21.0
Q1–Q3	19.0–25.0	19.0–22.0	18.0–24.0	17.5–24.0	19.0–24.0	21.0–22.0	20.0–22.0	18.0–24.0	19.0–24.0	17.0–23.5
Range	18–25	17–25	14–25	14–25	17–25	19–25	19–23	18–25	18–25	15–24
**Parent relation & home life**										

TH Open | DOI 10.1055/a-2781-8278 | © The Author(s).

Mean (SD)	26.1 (5.1)	25.1 (4.7)	25.5 (4.5)	24.8 (5.0)	24.8 (4.6)	26.4 (3.9)	27.0 (2.0)	25.0 (3.2)	25.3 (3.4)	27.0 (2.4)
Median	30.0	24.5	26.0	24.5	24.0	27.0	26.0	24.0	24.5	27.0
Q1–Q3	23.0–30.0	21.0–30.0	23.0–30.0	20.0–30.0	20.0–30.0	24.0–30.0	26.0–28.0	23.0–26.0	23.0–27.5	25.5–28.5
Range	16–30	17–30	18–30	16–30	18–30	21–30	25–30	22–30	22–30	24–30
**Social support & peers**										
Mean (SD)	21.4 (7.4)	24.2 (4.6)	22.6 (5.1)	22.2 (6.5)	25.4 (4.8)	26.6 (3.6)	27.0 (1.7)	26.4 (2.5)	27.0 (2.2)	24.3 (3.8)
Median	21.5	25.0	23.0	23.5	26.0	27.0	28.0	25.0	26.5	25.0
Q1–Q3	17.0–29.0	20.0–28.0	19.0–26.0	19.0–26.0	22.0–30.0	24.0–30.0	27.0–28.0	25.0–28.0	25.5–28.5	22.0–26.5
Range	6–30	17–30	15–30	6–30	15–30	22–30	24–28	24–30	25–30	19–28
**School environment**										
Mean (SD)	21.9 (7.4)	24.0 (3.3)	21.5 (8.4)	22.8 (6.4)	25.5 (3.9)	22.8 (5.6)	24.0 (6.4)	24.6 (5.9)	23.8 (7.2)	26.3 (4.8)
Median	24.0	24.0	25.0	23.5	26.0	21.0	25.0	28.0	27.0	27.5
Q1–Q3	19.0–28.0	22.0–25.0	17.0–28.0	20.0–28.0	23.0–29.0	20.0–27.0	22.0–29.0	21.0–29.0	19.5–28.0	22.5–30.0
Range	6–30	18–30	6–30	6–30	19–30	16–30	14–30	16–29	13–28	20–30
**Social acceptance (bullying)**										

TH Open | DOI 10.1055/a-2781-8278 | © The Author(s).

Mean (SD)	13.2 (2.6)	13.3 (2.2)	13.8 (1.8)	13.4 (2.0)	13.2 (2.0)	12.8 (2.5)	12.8 (2.5)	13.8 (1.6)	13.5 (2.4)	13.8 (1.9)
Median	14.5	14.0	15.0	14.5	14.0	13.0	13.0	14.0	14.5	14.5
Q1–Q3	12.0–15.0	12.0–15.0	13.0–15.0	12.0–15.0	12.0–15.0	12.0–15.0	12.0–15.0	14.0–15.0	12.0–15.0	12.5–15.0
Range	7–15	8–15	10–15	9–15	10–15	9–15	9–15	11–15	10–15	11–15
**Financial resources**										
Mean (SD)	10.1 (4.2)	10.8 (3.6)	10.6 (4.6)	10.7 (2.7)	11.3 (3.9)	7.6 (3.8)	9.0 (4.3)	9.8 (5.2)	7.3 (5.7)	9.8 (5.1)
Median	9.0	11.0	12.0	10.5	12.0	9.0	9.0	9.0	5.5	10.5
Q1–Q3	6.0–15.0	9.0–15.0	6.0–15.0	9.0–12.0	9.0–15.0	4.0–11.0	8.0–10.0	7.0–15.0	3.0–11.5	6.0–13.5
Range	3–15	4–15	3–15	6–15	3–15	3–11	3–15	3–15	3–15	3–15

The J-KIDSCREEN-52 questionnaire was only completed by participants aged 6 to <16 years old.

HA, haemophilia A; Q, quartile; QoL, quality of life; SD, standard deviation; Wk, week

TH Open | DOI 10.1055/a-2781-8278 | © The Author(s).

Supplementary Table 3D. J-KIDSCREEN-52 QoL results for all participants with absence (n=8) and presence (n=11) of bleeding at enrolment

QoL endpoints	Absence of bleeding					Presence of bleeding				
	Wk1	Wk25	Wk49	Wk73	Wk97	Wk1	Wk25	Wk49	Wk73	Wk97
**Number of participants (n)**	8	8	7	5	7	11	11	11	11	11
**Physical wellbeing**										
Mean (SD)	18.4 (4.3)	20.6 (2.0)	19.6 (4.0)	20.4 (4.4)	22.3 (1.6)	19.8 (3.1)	18.8 (3.7)	18.9 (4.0)	18.5 (3.7)	18.0 (4.4)
Median	18.0	20.0	20.0	21.0	22.0	20.0	17.0	19.0	18.0	19.0
Q1–Q3	17.0–21.5	20.0–21.0	16.0–23.0	21.0–22.0	21.0–23.0	17.0–21.0	16.0–21.0	16.0–23.0	15.0–21.0	15.0–22.0
Range	10–24	18–25	15–25	13–25	20–25	15–25	14–25	13–25	13–25	8–23
**Psychological wellbeing**										
Mean (SD)	25.8 (4.4)	26.6 (3.7)	25.6 (3.1)	22.6 (4.8)	28.1 (2.3)	25.6 (4.2)	25.7 (3.8)	24.7 (4.9)	24.9 (4.2)	25.3 (4.2)
Median	26.5	27.5	26.0	22.0	29.0	27.0	27.0	24.0	25.0	27.0
Q1–Q3	23.0–29.5	23.0–30.0	23.0–28.0	19.0–24.0	27.0–30.0	22.0–30.0	24.0–29.0	20.0–30.0	23.0–28.0	21.0–29.0
Range	18–30	22–30	21–30	18–30	24–30	18–30	19–30	18–30	15–30	20–30
**Moods & emotions**										
Mean (SD)	31.0 (5.0)	32.4 (4.4)	31.9 (3.3)	29.0 (5.3)	30.9 (5.0)	29.1 (3.8)	30.3 (3.4)	28.5 (3.5)	28.6 (4.4)	30.5 (3.5)
Median	33.0	34.0	33.0	28.0	33.0	28.0	30.0	28.0	28.0	30.0

TH Open | DOI 10.1055/a-2781-8278 | © The Author(s).

Q1–Q3	27.5–34.5	32.0–35.0	28.0–35.0	25.0–34.0	28.0–35.0	26.0–33.0	27.0–33.0	27.0–30.0	26.0–32.0	28.0–34.0
Range	23–35	22–35	27–35	23–35	21–35	25–35	25–35	22–35	20–35	24–35
**Self-perception**										
Mean (SD)	21.5 (2.3)	21.9 (2.2)	21.3 (3.6)	19.2 (3.5)	22.4 (1.8)	20.5 (3.0)	19.0 (3.8)	19.5 (2.6)	19.4 (3.1)	19.6 (2.7)
Q1–Q3	19.5–23.5	20.5–23.0	20.0–24.0	16.0–21.0	20.0–24.0	18.0–24.0	17.0–22.0	18.0–21.0	16.0–21.0	18.0–22.0
Median	21.0	22.5	22.0	21.0	23.0	20.0	18.0	19.0	20.0	20.0
Range	19–25	18–25	14–25	15–23	20–24	16–25	13–25	15–25	14–25	15–25
**Autonomy**										
Mean (SD)	22.3 (2.5)	22.0 (2.1)	22.7 (3.3)	22.0 (3.3)	22.7 (1.8)	21.2 (2.4)	20.4 (2.6)	19.0 (2.6)	19.6 (3.6)	19.9 (3.1)
Median	22.0	22.0	24.0	23.0	23.0	21.0	20.0	19.0	20.0	19.0
Q1–Q3	20.0–25.0	20.5–23.5	19.0–25.0	19.0–25.0	21.0–24.0	19.0–22.0	18.0–22.0	18.0–20.0	17.0–23.0	18.0–24.0
Range	19–25	19–25	17–25	18–25	20–25	18–25	17–25	14–25	14–25	15–25
**Parent relation & home life**										
Mean (SD)	26.5 (5.7)	26.9 (4.2)	26.1 (3.1)	24.4 (5.2)	25.7 (4.6)	25.9 (4.2)	24.6 (4.2)	24.9 (4.7)	25.1 (4.5)	25.0 (4.2)
Median	30.0	29.0	26.0	22.0	27.0	24.0	25.0	24.0	25.0	24.0
Q1–Q3	23.0–30.0	23.5–30.0	24.0–30.0	20.0–30.0	20.0–30.0	23.0–30.0	21.0–29.0	22.0–30.0	23.0–30.0	22.0–30.0
Range	16–30	20–30	22–30	20–30	20–30	19–30	17–30	18–30	16–30	18–30
**Social support & peers**										

TH Open | DOI 10.1055/a-2781-8278 | © The Author(s).

Mean (SD)	24.4 (8.2)	26.3 (3.7)	26.0 (2.2)	26.0 (3.3)	27.0 (2.4)	21.6 (5.9)	24.0 (4.4)	22.2 (5.5)	22.2 (6.7)	24.0 (5.2)
Median	28.5	28.0	25.0	27.0	27.0	22.0	26.0	20.0	24.0	23.0
Q1–Q3	21.5–29.5	24.0–28.5	24.0–28.0	25.0–27.0	25.0–30.0	17.0–27.0	20.0–28.0	17.0–29.0	19.0–26.0	20.0–30.0
Range	6–30	19–30	24–30	21–30	24–30	13–30	17–30	15–30	6–30	15–30
**School environment**										
Mean (SD)	22.1 (7.9)	24.5 (3.5)	25.9 (3.5)	23.2 (9.7)	27.3 (3.1)	22.2 (6.4)	23.6 (4.7)	20.1 (9.0)	22.9 (4.9)	24.6 (4.2)
Median	24.5	25.0	27.0	28.0	29.0	22.0	23.0	21.0	22.0	25.0
Q1–Q3	18.5–27.5	23.0–26.0	21.0–29.0	26.0–28.0	24.0–30.0	19.0–28.0	21.0–29.0	14.0–29.0	20.0–28.0	20.0–29.0
Range	6–30	18–30	21–29	6–28	23–30	8–30	14–30	6–30	13–30	19–30
**Social acceptance (bullying)**										
Mean (SD)	13.8 (2.8)	13.9 (1.6)	14.9 (0.4)	14.8 (0.4)	13.6 (2.1)	12.6 (2.2)	12.6 (2.5)	13.1 (1.9)	12.8 (2.2)	13.2 (1.9)
Median	15.0	15.0	15.0	15.0	15.0	13.0	13.0	13.0	13.0	13.0
Q1–Q3	14.0–15.0	12.5–15.0	15.0–15.0	15.0–15.0	11.0–15.0	11.0–15.0	11.0–15.0	11.0–15.0	11.0–15.0	12.0–15.0
Range	7–15	11–15	14–15	14–15	10–15	9–15	8–15	10–15	9–15	10–15
**Financial resources**										
Mean (SD)	8.9 (4.5)	10.5 (4.2)	11.6 (4.7)	10.4 (5.0)	11.6 (4.4)	9.9 (4.1)	10.2 (3.6)	9.6 (4.7)	9.5 (3.3)	10.5 (4.0)
Median	9.0	10.0	15.0	10.0	12.0	9.0	10.0	9.0	9.0	12.0
Q1–Q3	5.0–12.5	7.5–15.0	9.0–15.0	9.0–15.0	9.0–15.0	6.0–15.0	9.0–12.0	5.0–15.0	8.0–12.0	9.0–15.0

TH Open | DOI 10.1055/a-2781-8278 | © The Author(s).

Range	3–15	4–15	3–15	3–15	3–15	3–15	3–15	3–15	3–15	3–15

The J-KIDSCREEN-52 questionnaire was only completed by participants aged 6 to <16 years old.

Q, quartile; QoL, quality of life; SD, standard deviation; Wk, week

TH Open | DOI 10.1055/a-2781-8278 | © The Author(s).

Supplementary Table S4. J-KIDSCREEN-52 standardised T-scores for all participants aged 6–<16 years (n=19)

J-KIDSCREEN-52 Domain	Week 1	Week 25	Week 49	Week 73	Week 97
**Number of participants (n)**	19	19	18	16	18
**Physical wellbeing**					
Mean (SD)	50.1 (10.7)	51.3 (10.9)	51.1 (12.6)	50.0 (11.9)	50.6 (10.6)
Median (Q1–Q3)	47.1 (44.7–52.4)	49.6 (42.5–55.6)	48.4 (42.5–59.4)	49.6 (40.5–55.9)	51.0 (44.7–55.6)
Range	30.6–73.2	38.5–73.2	34.7–73.2	34.7–73.2	25.1–73.2
**Psychological wellbeing**					
Mean (SD)	54.1 (11.6)	54.8 (10.8)	52.2 (11.3)	50.0 (11.1)	55.7 (10.9)
Median (Q1–Q3)	54.5 (43.3–68.5)	54.5 (47.1–68.5)	49.5 (41.5–61.6)	47.1 (43.3–56.1)	56.1 (45.1–68.5)
Range	36.9–68.5	38.4–68.5	36.9–68.5	32.8–68.5	39.9–68.5
**Moods & emotions**					
Mean (SD)	52.9 (11.8)	56.1 (11.7)	51.8 (10.6)	50.3 (11.9)	54.3 (11.1)
Median (Q1–Q3)	51.3 (42.5–62.1)	57.4 (45.4–70.9)	47.3 (45.4–57.4)	45.4 (41.9–58.0)	52.7 (45.4–62.1)
Range	38.9–70.9	37.8–70.9	37.8–70.9	35.7–70.9	36.7–70.9
**Self-perception**					
Mean (SD)	52.3 (9.2)	50.7 (8.9)	50.4 (8.6)	48.1 (7.4)	51.1 (7.5)
Median (Q1–Q3)	47.8 (46.1–60.1)	49.8 (44.6–55.4)	47.8 (44.6–52.2)	47.8 (41.8–49.8)	47.8 (46.1–55.4)
Range	41.8–69.8	37.9–69.8	39.2–69.8	39.2–69.8	40.5–69.8

TH Open | DOI 10.1055/a-2781-8278 | © The Author(s).

**Autonomy**					
Mean (SD)	54.8 (9.0)	52.8 (8.0)	52.6 (10.3)	52.5 (10.6)	53.0 (8.5)
Median (Q1–Q3)	53.2 (46.9–68.8)	53.2 (46.9–53.2)	47.8 (45.1–60.5)	48.7 (45.2–62.5)	49.7 (46.9–60.5)
Range	45.2–68.8	43.6–68.8	39.0–68.8	39.0–68.8	40.5–68.8
**Parent relation & home life**					
Mean (SD)	55.0 (12.4)	52.2 (10.9)	51.7 (11.0)	50.9 (11.6)	51.6 (11.2)
Median (Q1–Q3)	65.9 (44.1–65.9)	49.5 (41.1–65.9)	47.6 (44.1–65.9)	46.6 (41.1–65.9)	48.8 (42.6–65.9)
Range	34.3–65.9	35.7–65.9	37.0–65.9	34.3–65.9	37.0–65.9
**Social support & peers**					
Mean (SD)	50.0 (16.5)	52.8 (9.9)	50.9 (11.7)	49.7 (15.1)	54.8 (12.3)
Median (Q1–Q3)	48.4 (39.5–62.7)	52.4 (45.1–58.1)	48.4 (42.2–58.1)	49.3 (42.2–54.9)	50.2 (45.1–71.5)
Range	9.4–71.5	38.2–71.5	35.4–71.5	9.4–71.5	35.4–71.5
**School environment**					
Mean (SD)	50.8 (15.1)	54.2 (9.8)	50.9 (17.1)	51.8 (13.9)	59.0 (11.3)
Median (Q1–Q3)	52.2 (43.8–61.9)	52.2 (48.6–58.9)	55.3 (40.9–65.9)	54.2 (45.3–61.9)	56.6 (50.4–73.8)
Range	14.0–73.8	36.8–73.8	14.0–73.8	14.0–73.8	43.8–73.8
**Social acceptance (bullying)**					
Mean (SD)	48.0 (11.7)	47.7 (11.4)	50.6 (10.2)	49.1 (11.0)	48.4 (11.2)
Median (Q1–Q3)	48.1 (38.3–58.9)	42.2 (38.3–58.85)	58.9 (42.2–58.9)	53.5 (38.3–58.9)	53.5 (38.3–58.9)

TH Open | DOI 10.1055/a-2781-8278 | © The Author(s).

Range	27.2–58.9	29.1–58.9	33.1–58.9	31.1–58.9	33.1–58.9
**Financial resources**					
Mean (SD)	44.2 (13.0)	46.6 (11.9)	47.5 (14.8)	44.6 (12.0)	48.3 (13.2)
Median (Q1–Q3)	41.9 (35.1–56.4)	44.2 (39.7–62.9)	45.6 (35.1–62.9)	43.1 (39.7–49.3)	49.3 (41.9–62.9)
Range	23.2–62.9	23.2–62.9	23.2–62.9	23.2–62.9	23.2–62.9

For participants who were unable to enter data themselves, the caregiver of the participant assisted with completing the questionnaire based on the responses of the participant. The J-KIDSCREEN-52 questionnaire was only completed by participants aged 6 to <16 years old.

Q, quartile; SD, standard deviation

TH Open | DOI 10.1055/a-2781-8278 | © The Author(s).

Supplementary Table S5. Daily life questionnaire caregivers' comments before and after beginning emicizumab (n=39)

Age of child, years	Analysis visit	Caregiver relationship to child	Change in activity	Change in motivation for work/school	Change in anxiety about bleeding	Comments
9	Wk97	Mother	Increased	Proactive	Decrease	Since my child has grown and I have almost no concerns about bleeding. I have change my job from a part-time job to a full-time employee. It has became easier to inject once a week, and I had no anxiety.
0	Wk49	Mother	Slightly increased	No change	Slightly decreased	I now have less concern about bleeding and can have my brother play in the same space as my brother.
3	Wk97	Mother	Increased	Proactive	Slightly increased	Fortunately, there were no major injuries, but I still feel anxious all the time.
1	Wk49	Mother	No change	No change	Slightly decreased	I'm less concerned about injury as a parent. Even when mild subcutaneous bleeding occurs, I feel that it heals quickly.

TH Open | DOI 10.1055/a-2781-8278 | © The Author(s).

2	Wk49	Mother	No change	No change	Slightly decreased	When the wound was formed or injected, it was swollen right away and the blood did not go away, but after the injection of a hebryler, the frequency decreased and I can play as much as I can I think.
	Wk97	Mother	Slightly increased	No change	Decrease	After I started using Hemliblra, the frequency of bruising obviously decreased, and I feel like it does not take long to bleed. I started a gymnastic class, and I am less concerned about restricted exercise.
0	Wk49	Mother	Increased	Proactive	Slightly decreased	Subcutaneous bleeding dramatically decreased compared to before administration of Hemlibra, and it seems great.
	Wk97	Mother	Increased	Proactive	Slightly decreased	Compared to my brother, he is active, and bleeding is sometimes suspected, but probably because he is receiving Hemlibra, he needs to stop bleeding immediately. It would be nice if he could continue playing and having fun with his friends on his own will.
4	Wk49	Mother	Increased	Proactive	Decrease	After I started Hemlibra, I was able to play freely, and I felt extremely comfortable emotionally.

TH Open | DOI 10.1055/a-2781-8278 | © The Author(s).

	Wk97	Mother	Slightly increased	No change	Decrease	The constant bruises on the legs and arms have disappeared, and joint swelling has greatly decreased. I can now make them stop doing things.
1	Wk49	Mother	Increased	Proactive	Slightly decreased	Before learning about Hemlibra. I was only anxious about visiting the hospital multiple times a week to receive injections of factor 8 and worried about whether to continue working. When I received the injection for the first time, it was difficult to secure a route, and the repeated needle sticks I received doubled my anxiety. It was decided that I would consult with the doctor about regular vaccination with Hemlibra at the time of my return to work from maternity leave, and the doctor start it. Now, I live far from the way I had imagined, in a good sense, and I can live my life without any major anxiety. I appreciate it.
	Wk97	Mother	Increased	Proactive	Decrease	Before I started Hemlibra, I use to tend to limit what I could do if I fell and hit myself, but now I can get used to not restricting myself too much, saying I don’t need to panic or be OK if I hit myself.

TH Open | DOI 10.1055/a-2781-8278 | © The Author(s).

0	Wk49	Father	No change	No change	Decrease	Before I started Hemlibra, I was always anxious about the risk of bleeding. Even if I move around actively with Hemlibra, I don’t feel anxious. In addition, in the past, patients were urgently transported to the hospital due to sudden haemorrhage and were injected with the drug product, but now, there are none, and it has become very easy to spend my daily life.
1	Wk49	Mother	Increased	Proactive	Decrease	I can now fully enjoy myself in the park. From immediately after the first treatment, the bruises decreased, and I felt a great effect.
	Wk97	Mother	Increased	Proactive	Decrease	Even when bleeding occurred after the start of treatment, it stopped immediately and the bruises also decreased, so I felt the effects of treatment. Thanks to this, I was able to encourage them to try various things like other children!
0	Wk49	Mother	No change	No change	Slightly decreased	Since I started using Hemlibra, I am relieved that my bruises have decreased a great deal, but I feel anxious when I get a big injury or it my head hard.

TH Open | DOI 10.1055/a-2781-8278 | © The Author(s).

	Wk97	Mother	Increased	No change	Slightly decreased	At first, I was anxious, but even when I got bigger and started to move more actively, there was no noticeable bleeding, so I am at ease to some extent.
0	Wk97	Mother	No change	No change	Slightly decreased	I was able to expand my range of activities by giving injections. Slides, etc.
2	Wk49	Mother	No change	No change	Decrease	After the launch of Hemlibra, it became difficult to develop bruises etc.
0	Wk49	Mother	No change	No change	Slightly decreased	Since it is still small, there is not much change in my life. I started Hemlibra injection without causing major bleeding. Since I have not been injured yet, I do not feel any effect, but I feel a sense of security that I am receiving Hemlibra.
	Wk97	Mother	No change	No change	Slightly decreased	The administration of Hemlibra slightly alleviated the child's anxiety about exercise.

Comments collected at Week 49 and 97 at discontinuation of the study. Comments from caregivers in this table were provided in Japanese and directly translated to English, which does not account for potential syntax, punctuation and grammatical errors.

Wk, week.

TH Open | DOI 10.1055/a-2781-8278 | © The Author(s).
